# Microglia Response During Parkinson’s Disease: Alpha-Synuclein Intervention

**DOI:** 10.3389/fncel.2018.00247

**Published:** 2018-08-06

**Authors:** Sara A. Ferreira, Marina Romero-Ramos

**Affiliations:** ^1^AU IDEAS center NEURODIN, Aarhus University, Aarhus, Denmark; ^2^Danish Research Institute of Translational Neuroscience - DANDRITE, Nordic-EMBL Partnership for Molecular Medicine, Aarhus University, Aarhus, Denmark; ^3^Department of Biomedicine, Aarhus University, Aarhus, Denmark

**Keywords:** neuroinflammation, toll-like-receptors, integrins, neurodegeneration, T-cells, MHC-II, DAMP, sterile inflammation

## Abstract

The discovery of the central role played by the protein alpha-synuclein in Parkinson’s disease and other Lewy body brain disorders has had a great relevance in the understanding of the degenerative process occurring in these diseases. In addition, during the last two decades, the evidence suggesting an immune response in Parkinson’s disease patients have multiplied. The role of the immune system in the disease is supported by data from genetic studies and patients, as well as from laboratory animal models and cell cultures. In the immune response, the microglia, the immune cell of the brain, will have a determinant role. Interestingly, alpha-synuclein is suggested to have a central function not only in the neuronal events occurring in Parkinson’s disease, but also in the immune response during the disease. Numerous studies have shown that alpha-synuclein can act directly on immune cells, such as microglia in brain, initiating a sterile response that will have consequences for the neuronal health and that could also translate in a peripheral immune response. In parallel, microglia should also act clearing alpha-synuclein thus avoiding an overabundance of the protein, which is crucial to the disease progression. Therefore, the microglia response in each moment will have significant consequences for the neuronal fate. Here we will review the literature addressing the microglia response in Parkinson’s disease with an especial focus on the protein alpha-synuclein. We will also reflect upon the limitations of the studies carried so far and in the therapeutic possibilities opened based on these recent findings.

## Introduction

Parkinson’s disease (PD) is the second most common neurodegenerative disease and it is characterized by the loss of dopaminergic neurons in substantia nigra and the presence of intra neuronal cytoplasmic inclusions composed mainly of aggregated alpha-synuclein (a-syn) named Lewy bodies (LB) and neurites. The protein a-syn plays a central role in the pathogenesis of the disease since multiplication or mutations of the gene are associated with familial forms of PD (Koros et al., 2017). A-syn is suggested to exist as an unfolded monomeric form, although a controversial tetrameric form has been also suggested to exist, stabilizing the protein (Villar-Pique et al., 2016). During PD, a-syn progressively transforms from soluble to insoluble fibrillary complexes, most likely through an intermediate soluble oligomeric form (Cremades et al., 2017) leading to neuronal dysfunction (Eschbach and Danzer, 2014), where the dopaminergic neurons are the most susceptible and will degenerate and die. As a consequence of the nigral cell loss, dopamine levels would decrease in caudate-putamen leading to a malfunction of the basal ganglia circuitry (Obeso et al., 2008). This results in the four cardinal symptoms of PD: resting tremor, bradykinesia, muscle rigidity, and postural instability (Gopalakrishna and Alexander, 2015). Although, these motor symptoms are the hallmark of PD, they are preceded by other non-motor symptoms, which are not directly related to the dopamine loss but that seem correlated to a-syn pathology in other neuronal populations, such as constipation, hyposmia, and rapid eye movement (REM)-sleep behavior disorder (Schapira et al., 2017). Furthermore, cognition is also impaired in PD and the progression and worsening of these symptoms results in dementia at later stages of PD (Aarsland et al., 2017).

As mentioned, the pathological a-syn accumulation in the different neuronal populations in brain of patients seems to be associated to all main symptoms in PD, therefore suggesting that aggregated a-syn leads to neuronal dysfunction. This was highlighted by Braak and Braak’s work in post-mortem PD brains which resulted in their proposal of the staging scheme in PD, based on their correlation between a-syn pathology and PD progression (Braak et al., 2003). According to this scheme, in stage 1 a-syn pathology is present in the olfactory nucleus and dorsal motor nucleus of the vagus nerve, which would explain why enteric system and other autonomic dysfunction, together with hyposmia, appear as early symptoms in PD. Stage 2 shows a-syn pathology in medulla oblongata and the pontine tegmentum with raphe and locus coeruleus affected which are related to symptoms of depression (Boeve et al., 2007). It is not only until stage 3 that a-syn pathology is found in substantia nigra and amygdala. This stage is also considered a pre-symptomatic phase, where the REM sleep disorder is suggested as a manifest pre-motor symptom (Boeve et al., 2007). At certain time on this stage, PD patients experience the first motor symptoms. The feature of stage 4, is associated with the anteromedial temporal mesocortex and it is here that overt parkinsonism is evident. The last two stages, 5 and 6, involve the entire neocortex and it is at this stage that PD patients manifest the full range of clinical symptoms, where cognition problems are the most remarkable (Braak et al., 2003).

Neurons constitutively synthesized a-syn and the protein is normally found in synapses. The protein can adopt certain structure upon interaction with lipids and it has been suggested to be involved in vesicle homeostasis (review in Burre, 2015). The neurons release a-syn to the extracellular space through an exocytosis process. This happens for all forms of the protein: soluble or misfolded (Emmanouilidou and Vekrellis, 2016). Recent findings suggest that the a-syn neuronal pathology can spread from one cell to another. Indeed, the presence of LB in fetal transplanted neurons in PD patients found in post-mortem analysis over a decade after they received the transplants (Kordower et al., 2008; Li et al., 2008), suggested that otherwise healthy neurons could uptake misfolded a-syn from the neighboring neurons, and that this host aggregated a-syn could act as a seed accelerating the aggregation of the a-syn in the healthy fetal transplanted cell (Tyson et al., 2016). This hypothesis was also supported by *post-mortem* studies from Braak & Braak suggesting that a-syn pathology in PD may start in neurons of the peripheral nervous system (gut or nose) to later propagate progressively to anatomically connected regions of the CNS, spreading in a prion-like manner (Braak et al., 2003). The hypothesis has been later sustained by different findings in animal models, where injections of extracellular aggregated a-syn lead to a-syn pathology in neurons in the vicinity of the injection, but also in anatomically related neurons (Luk et al., 2012; Rey et al., 2013). However, a lack of consistency of the hypothesis in the brains of human patients has been discussed and the hypothesis remains controversial (Brundin and Melki, 2017; Surmeier et al., 2017). Despite this, the acceptance that a-syn pathology is found in multiple neuronal populations has highlighted the role of other areas different than the substantia nigra in the disease introducing the concept of PD as a multisystem disorder. In the disease, the mishandling of a-syn is accepted to lead to multiple dysfunction that will promote neurodegeneration with or without cell death. Several mechanisms, such autophagy failure, mitochondria dysfunction, endoplasmic reticulum stress and calcium dysregulation have been proposed to be responsible of the a-syn induced neurodegeneration; we refer the readers somewhere else for further information (Benskey et al., 2016; Wong and Krainc, 2017). In this review, we will focus our attention on the role of microglia in the neurodegenerative process of PD, with a special emphasis in the protein a-syn.

## Immune Response in CNS During Parkinson’s Disease: the Microglia

In the last decade it has become apparent that in parallel to the a-syn induced neuronal process, inflammatory changes in brain and periphery also occur in PD patients (Kannarkat et al., 2013). It is suggested the chronic inflammatory process contributes to neuronal degeneration via overproduction of pro-inflammatory cytokines and oxidative stress. Accordingly, the relevance of the immune process has been supported by the epidemiological data suggesting a protective effect for the NSAID in humans (Gagne and Power, 2010; Gao et al., 2011). Within the brain, the microglia will be the main instructor of such response. Microglia arise from embryonic yolk sac precursors, different to those generating the hematopoietic monocytic lineage, thus constituting different cells than bone marrow derived macrophages (Li and Barres, 2017). They have been shown, using single cell genetic analysis, to undergo stepwise genetic stages in synchrony with neuronal changes during brain development, although they are also influenced by peripheral changes such as maternal microbiota (Matcovitch-Natan et al., 2016). Microglia are continuously monitoring their micro-environment and are able to sense changes in the brain (Nimmerjahn et al., 2005). They possess receptors for molecules of immune and also neuronal origin, such as neurotransmitters; hence, microglia are able to monitor neuronal as well as immune activity (Biber et al., 2007; Kettenmann et al., 2011). They are shown to be essential for synaptic remodeling via trogocytosis of axonal structures (Weinhard et al., 2018).

The term microglia activation or microgliosis, comprises any deviation from what otherwise is considered normal microglia status, including cell number, morphology or protein expression (Joers et al., 2016). Microgliosis, has been shown in PD *post-mortem* studies and also most recently on *in vivo* PET imaging analysis of prodromal (REM sleep disorder patients) and diagnosed PD patients (Gerhard et al., 2006; Stokholm et al., 2017). The presence of numerous MHCII+ cells in the brain of PD patients has been shown in several occasions (McGeer et al., 1988; Croisier et al., 2005; Orr et al., 2005; Miklossy et al., 2006). MHCII, or in fact HLA expression, is normally very low in brain and it is related of the antigen presenting system in myeloid cells. Interestingly HLA polymorphism have been related to increased risk of develop PD in GWAS studies (Nalls et al., 2014). In addition, other markers are also shown upregulated in microglia in patients: such as pro-inflammatory enzymes iNOS and COX (Hunot et al., 1996; Knott et al., 2000) and CD68, usually associated to phagocytic activity (Banati et al., 1998; Croisier et al., 2005; Doorn et al., 2014). Moreover, increase pro-inflammatory cytokines have been shown in human PD brain, such as IL-1β, IL-2, IL-6, EGF, and TGF-α and TGF-β in striatum (Nagatsu et al., 2000) and TNF-α upregulation in nigral microglia (Boka et al., 1994), altogether supporting a chronic pro-inflammatory milieu in the brain of PD patients.

Besides MHCII, other markers further suggest the cross-talk of the microglia with the adaptive immune system, such as the increase in lymphocyte function associated protein LFA1 (CD11a) (Imamura et al., 2003a; Miklossy et al., 2006), and its receptor: the intercellular adhesion molecule 1, ICAM1 (CD54) (Imamura et al., 2003a). Also, the increased expression of FcγR in microglia that was found in the vicinity of neurons with IgG deposition (Orr et al., 2005) supporting a role for the humoral response in the disease. The common non-coding HLA single nucleotide polymorphism associated to increased PD risk, *rs3129882*, has been associated to a shift toward a pro-inflammatory CD4+ T cell response upon certain environmental exposures (Kannarkat et al., 2015), suggesting the involvement of lymphocytes in the disease. In fact, infiltrated T-cells are present in *post-mortem* PD human brains (McGeer et al., 1988; Brochard et al., 2009). Within the adaptive immunity, the protein a-syn seems to play a key role and actually, HLA+ microglia is correlated with a-syn neuronal deposition (Croisier et al., 2005). In addition, decreased levels of autoantibodies against a-syn have been described in PD patients, suggesting a protective effect for these antibodies (Brudek et al., 2017). Furthermore, Sulzer et al., has recently shown that PD patients’ derived T-cells exhibited mostly a CD4+ Th2 response (IL-5) related to a-syn peptides, further suggesting that the immune system responds and can act as a protector of neuronal events related to a-syn (Sulzer et al., 2017).

The evidence that the immune response in PD is not restricted to microglia in brain, is multiplying during the last decade. The changes in peripheral monocytes (Funk et al., 2013; Grozdanov et al., 2014), lymphocytes (Bas et al., 2001; Gruden et al., 2011; Stevens et al., 2012) as well as changes in immune soluble mediators are numerous now. We will however, do not review this immune aspect in this occasion and refer the reader to our prior work (Romero-Ramos, 2017).

## Microgliosis as a Marker of Neurodegeneration

Multiple *post-mortem* analysis revealed that microgliosis occurs in all the areas where a-syn and neurodegeneration happen, irrespective of the presence or absence of cell death (Hunot et al., 1996; Knott et al., 2000; Imamura et al., 2003b; Croisier et al., 2005; Halliday and Stevens, 2011; Doorn et al., 2014). Moreover, *in vivo* analysis of microglia activation using positron emission tomography (PET) in patients, suggest that the activation is an early but sustained response in PD, not limited only to the areas with significant neuronal death. Microglia activation in midbrain seems to occur very early and it is consistently found in most studies. PET imaging of the peripheral benzodiazepine receptor (PBR/TSPO) binding ligand [11C]-(R) PK11195, revealed microglial activation in the midbrain of patients with REM sleep disorders (prodromal PD) (Stokholm et al., 2017). The microgliosis progresses to be found later in other areas in already diagnosed PD patients such as putamen (Ouchi et al., 2005; Iannaccone et al., 2013), hippocampus (Doorn et al., 2014), and cortex (Imamura et al., 2003a). These areas are also highlighted by [11C]-(R) PK11195 PET of patients, such as pons and cortex (Gerhard et al., 2006). This stage progression could explain why some *post-mortem* studies observed activation of microglia in the substantia nigra but not in striatum due to a mix cohort of patients in different stages (Mirza et al., 2000). Altogether, evidence suggests that the response of microglia is early and not related only to cell death but rather to a-syn pathology, supporting activated microglia as a sensitive index of neuropathological changes (Imamura et al., 2003a). The presence of microgliosis first in midbrain, might be related to the higher susceptibility of this area in PD (Surmeier et al., 2012) and also to the described higher density of microglia in substantia nigra (Lawson et al., 1990; Yang et al., 2013). In fact, flow cytometry and recently single cell gene analysis, have shown that adult brain microglia differ from one brain area to another (de Haas et al., 2008; Matcovitch-Natan et al., 2016); probably as a response to the environment, which is given among other things by the phenotype of the neuronal input and activity in the area and by the type of a-syn aggregation found at each stage. In fact, we and others have postulated that the microglia response during PD is dynamic and that such response will have consequences in the neuronal fate (Sanchez-Guajardo et al., 2010; Moehle and West, 2015).

## Microgliosis in Animal Models of Parkinsonian-Like Neurodegeneration

Multiple studies have shown that all classic models of PD-like neurodegeneration present some type of microgliosis. Accordingly, microglia activation is associated to toxic models of dopaminergic neurodegeneration such as 6-OHDA (Marinova-Mutafchieva et al., 2009; Walsh et al., 2011), MPTP (Wu et al., 2002; Smeyne et al., 2016; Manocha et al., 2017), rotenone (Ojha et al., 2016), and also in non-toxic such as the axotomized models (Cho et al., 2006). The relevance of the microgliosis for the neuronal fate is supported by those attempts to achieve neuroprotection using anti-inflammatory therapies but also, by the PD-models based on overt immune activation such as injections of LPS in the brain (Machado et al., 2011; Hoban et al., 2013). Thus, robust immune activation can lead to cell death, however, the opposite is also true and cell death *per se* can induce microgliosis. Upon cell death, certain proteins can be released from the dying neurons that will initiate a sterile inflammatory response (an immune response in the absence of pathogen, reviewed in Thundyil and Lim, 2015). This response will involve receptors normally express by microglia such as TLR and others (Rock et al., 2010). The process is initiated by one or more cellular components that possess intrinsic proinflammatory activity, thus they would act as DAMPs (damage-associated molecular patterns), such as the high mobility group box 1 (HMGB1), HSP60, ATP, mitochondrial peptides, and DNA (Rock and Kono, 2008). Accordingly, 6-OHDA induced neurotoxicity increases HSP60 levels (Feng et al., 2013). HMGB1, also related to sterile inflammation in brain, interacts with CD11b in microglia (Fang et al., 2012) and it has been shown to bind a-syn (Lindersson et al., 2004). In addition, release of ATP during cell death mediates microglia activation and chemotaxis response via purinergic receptors (Davalos et al., 2005). Thus, cell death will have consequences in the microglia response, further contributing to the immune response in the brain.

Nevertheless, the occurrence of cell death is not a necessary event for the activation of microglia in the brain. This has become especially apparent in protein-based models of PD focused on a-syn. In transgenic lines where dopaminergic neuronal death was occurring, like the a-syn A53T under the prion protein promoter (PrP) or the double mutant (DM) A30P+A53T a-syn under the TH promoter, microgliosis and changes in the expression of multiple immune related genes were observed to precede the cell death (Lee et al., 2002; Miller et al., 2007; Su et al., 2009). Moreover, mice overexpressing a-syn under TH promoter show very early microgliosis and up-regulation of TNF-α despite the absence of cell death (Su et al., 2008). Equally microgliosis in the substantia nigra and striatum of the Thy-1 wild type a-syn line preceded motor deficits and occurred despite the absence of neuronal death (Watson et al., 2012). Therefore, microgliosis is rather associated to a-syn pathology and neurodegeneration, without cell death, as it has been also described in other lines: like that overexpressing a C-terminal truncated α-syn (Tofaris et al., 2006), the A30P over-expressing mice under PrP (Gomez-Isla et al., 2003), and the E46K under the PrP (Emmer et al., 2011). We and others have also shown that the overexpression of a-syn by means of viral vectors will lead to early microgliosis that will be dynamic and will be influenced but not dependent of cell death (Theodore et al., 2008; Sanchez-Guajardo et al., 2010). This was also seen across species, as we have shown in non-human primates (Barkholt et al., 2012).

Within this microglia activation, many are the possible proteins and processes involved. Actually, microglia and neurons interact through a series of proteins that are involved in microglia activation (Kierdorf and Prinz, 2013). Microglia is continuously sensing neuronal activity and it helps reshaping neurons, synapse and circuits (Wake et al., 2013). Moreover, microglia are involved in neuronal transmission and *vice versa*, neuronal activity influences microglia activity and mobility (Li et al., 2012; Eyo and Wu, 2013; Dissing-Olesen et al., 2014). Thus, any change in the neurons during PD- changes in neurotransmitter release, changes in ATP production, synaptic loss, etc.- will be sensed by microglia, and subsequently initiates a response, i.e., microglia activation. We will focus on this review in the specific role of a-syn as an initiator of inflammation and in parallel in the role of microglia in the neurodegenerative process associated to a-syn.

## Microglia as a Phagocytic Cell Clearing Alpha-Synuclein

A-syn is released from the neurons in a calcium dependent manner (Emmanouilidou et al., 2010), so it can be found in CSF and extracellular space, as well as conditioned media of neurons expressing a-syn. This release seems to be related and dependent on neuronal firing (Emmanouilidou et al., 2016; Yamada and Iwatsubo, 2018). The release of a-syn seems to be crucial for the so-called spreading of a-syn, and, in this context, microglia would be highly relevant. First: microglia senses and modulates neuronal activity, approaching highly active neurons in brain (York et al., 2017). Furthermore, a-syn seems to act as a chemoattractant and direct microglia migration. The Membrane Type 1-MMP has been related to such chemoattractant ability, which was mediated by an increase in soluble CD44 (cell adhesion molecule) that frees microglia from surrounding matrix to migrate (Kim et al., 2009). In addition, a-syn interaction with CD11b and subsequent NOX2 activation, leads to increase H_2_O_2_, which can also act as a final direct signal for migration (Wang Q. et al., 2015). This is in agreement with the *post-mortem* study showing that in PD patients the activated microglia are in close contact with neurons presenting a-syn pathological accumulation (Croisier et al., 2005). However, it should be noted that microglia can and will encounter a-syn by the trogocytosis of neuronal structures as well (Weinhard et al., 2018).

Secondly: microglia will be the main cell clearing extracellular a-syn (Lee et al., 2008). This clearance process is influenced by the level of activation, and as well by the type of a-syn encountered (Lee et al., 2008; Park et al., 2008). Different proteins have been suggested to be involved in this uptake and clearance system and in the activation of microglia mediated by a-syn (see below). But also some extracellular enzymes have been suggested as responsible of the degradation of a-syn: metalloproteinase (Sung et al., 2005), neurosin (Tatebe et al., 2010), and plasmin (Kim et al., 2012), hence they could be relevant in the a-syn spreading and the immune response. Otherwise, extracellular a-syn should be degraded by microglia and this will avoid the spreading of the pathology to neighboring neurons. To add complexity to this, a-syn *per se* can modify the microglia activity and its functional capacity. Indeed, a-syn can act as DAMP and induce a sterile response that can lead to activation of microglia. This activation is dependent in the type and solubility of the a-syn and will lead to neurotoxicity (Zhang et al., 2005; Jin et al., 2007). In parallel, the a-syn induced activation, can lead in turn to production of reactive oxygen species (ROS) and oxidation of a-syn in neighboring neurons, which will feed the disease process (Shavali et al., 2006). Thus, the microglia response will evolve during PD as the neuronal function and type of a-syn progressively change. The response of microglia will in turn translate in immune signaling and neuronal changes that will have relevant consequences in the disease progression. To further increase the complexity, aging can be another factor in this equation, since microglia (and macrophages) ability to phagocyte monomeric and oligomeric a-syn decreases with age (Bliederhaeuser et al., 2016). Accordingly, telomerase shortening, a process associated to aging, has been shown to accelerate a-syn pathology *in vivo* in an a-syn transgenic mice that was associated to an impaired microglia immune response (Scheffold et al., 2016).

As mentioned, microglia will be the cell by default clearing a-syn in the extracellular space, but in parallel, a-syn will lead to microglia activation, though such a-syn mediated activation seems to be independent of internalization and phagocytosis (Zhang et al., 2007); even if they might affect each other at the long term. The literature regarding the phagocytic activity upon a-syn stimulation includes contradictory results, which can be due to the types of a-syn used, concentrations and cell-models of choice. One study has suggested distinct capacity associated to disease related mutations: while monomeric WT or A53T a-syn increased phagocytosis, A30P and E46K monomeric, decreased it (Roodveldt et al., 2010). Also Park et al. (2008) observed increased phagocytosis upon activation with monomeric a-syn that was not mediated by CD36, α6β1 integrin and CD47 receptor complex, but that was related to the N-terminal and NAC region of a-syn. The same team showed, however, that the phagocytosis was decreased if exposed to aggregated a-syn (Park et al., 2008). Accordingly, aggregated a-syn has been shown to inhibit microglial phagocytosis by activating SHP-1 via interaction with FcγRIIB (and upregulation of its expression) (Choi et al., 2015; York et al., 2017). Others reported no difference in the ability of the BV2 cells to phagocyte a-syn with respect to the solubility (monomeric, truncated or fibrillar) (Fellner et al., 2013), which they suggested was a TLR-4-dependent mechanism (Stefanova et al., 2011). Thus, yet unclear, this could suggest that certain molecular species of a-syn may interfere with the phagocytic ability of microglia.

Monocytes, lymphocytes and other immune cells express a-syn (Shin et al., 2000) and several studies have investigated the role of a-syn in immune response using a-syn knockout mice. The absence of a-syn resulted on impaired B cell (Xiao et al., 2014) and T-cells function (Shameli et al., 2016). Furthermore, the lack of a-syn in microglia results in exaggerated response to LPS and decreased phagocytic ability (Austin et al., 2006), that was related to excess activation of Phospholipase D2 and COX-2, suggesting that a-syn is normally needed for lipid mediated signaling by microglia (Austin et al., 2011). In the opposite, if overexpressed, a-syn in BV2 cells increased COX-2 levels and TNF-α secretion while the phagocytic activity is impaired (Rojanathammanee et al., 2011). A recent study using iPSC derived macrophages showed that triplication of SNCA gene leads to dysfunction in phagocytosis and such effect could be mimicked by the excess of extracellular a-syn in culture (Haenseler et al., 2017). PD human lymphocytes show a higher intracellular concentration of a-syn (Gardai et al., 2013), which might be related to the reported interference of pathological a-syn in the SNARE complex (Choi et al., 2013). Accordingly, PD derived lymphocytes and peripheral monocytes/macrophages from the BAC a-syn transgenic mouse line, showed impaired phagocytosis and cytokine release, due to defective vesicle transport, as a result of SNARE deficiency (Gardai et al., 2013). In a recent report, lack of a-syn in neurons lead to MHC-I increase expression in neurons and microglia activation suggesting a role for a-syn in the maintenance of a healthy immune balance in brain (Benskey et al., 2018). Therefore, both: loss and gain of function regarding a-syn can have important consequences in the ability of microglia to phagocyte and handle the protein maintaining a healthy CNS microenvironment in the process.

Regarding, the microglial internalization of a-syn, uptake of monomeric a-syn was reported to be independent of clathrin-, caveolae-, and dynamin, but rather dependent on GM-1-enriched membrane lipid rafts (Park et al., 2009). However, internalization of aggregated a-syn has been related to clathrin and calnexin (Liu et al., 2007). Different studies suggest that a-syn uptake is dependent on the aggregation status, increasing as the fibrilization growths (Hoffmann et al., 2016); or that the proteins responsible of the uptake differ for the different types of a-syn, as only oligomeric a-syn uptake has been shown to be TLR-2 mediated (Kim et al., 2013), while, the reported TLR-4 mediated a-syn uptake does not depend on the aggregation status (Stefanova et al., 2011). Galectin 3 has also been related to aggregated a-syn induced microglia activation and the a-syn induced increase of phagocytosis (Boza-Serrano et al., 2014). In relation to the subsequent degradation in microglia as in neurons, autophagy seems to play an important role (Nash et al., 2017), and in BV2 cells, oligomeric a-syn degradation was mediated through NRAMP1, a lysosomal iron transporter (Wu et al., 2017). However, in iPSC derived macrophages (not microglia) both lysosomal and proteasomal systems have been related to degradation of monomeric a-syn (Haenseler et al., 2017). Remarkably, deficiency of DJ-1, another protein related to genetic PD, reduces the levels of membrane lipid rafts and simultaneously limits the internalization of extracellular a-syn. Furthermore, the DJ1 deficiency also lead to the decrease in the ability to degrade a-syn by autophagy (Nash et al., 2017). Interestingly, while the TLR-4 has been related to increase a-syn uptake and clearance in microglia, the TLR-2 activation in neurons seems, on the contrary, to decrease the uptake and autophagy of a-syn, promoting neuronal a-syn accumulation (Kim et al., 2015). Actually, increased TLR-2 in neurons has been observed in PD human brains, and such increased was correlated to disease duration and pathological a-syn levels (Dzamko et al., 2017). Moreover, a-syn interaction with TLR-2 would induced a pro-inflammatory response both in microglia and also in neurons (Kim et al., 2013; Dzamko et al., 2017). Thus, signaling cascade initiated by the same receptor in different cell types will contribute differently to the disease.

**FIGURE 1:**
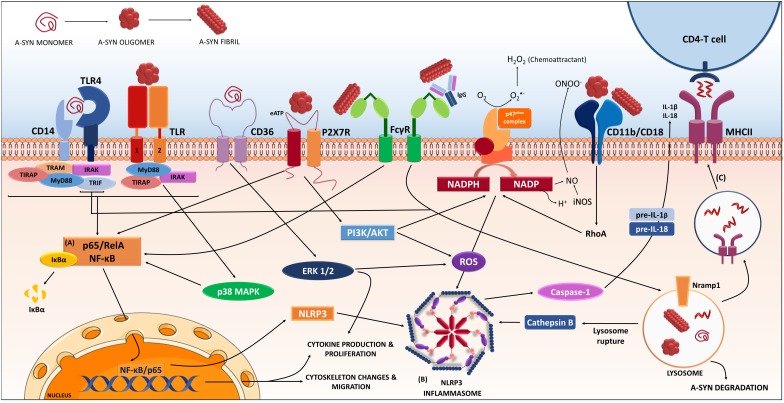
Receptors and proteins involved in the a-synuclein induced response in microglia. The microglia will respond to a-synuclein that might be encountering upon trogocytosis of synapses, but also in the extracellular milieu via exocytosis from neighboring neurons. The type of a-synuclein will change as the disease progresses, thus microglia will encounter, i.e., monomeric, oligomeric, or fibrillar a-synuclein, but also other form such as nitrated, truncated, and phosphorylated a-synuclein (not shown). In addition, a-synuclein might be complexed to other molecules such as IgG. The microglia will recognize, uptake and phagocyte the different a-synucleins, in a process that will be dependent on the type of a-synuclein encountered and the proteins and cascades involved in such event might differ accordingly. Data from multiple labs support the pro-inflammatory character of the event initiated by a-synuclein and thus its ability to act as a DAMP. Such event is the consequence of the interaction of a-synuclein with membrane receptors that lead to the **(A)** NF-κB activation through several identified mediators, and to **(B)** assembly of the NLRP3 inflammasome. Altogether, results in the production of inflammatory mediators and free radicals. In addition, proliferation and migration will also occur as a result of these cascades. The microglia will also degrade a-synuclein in a process that, if compromised, can lead to further inflammatory signaling. In parallel the microglia can act as an antigen presenting cell and through the MHC-II system present a-synuclein peptides to CD4-T-cells **(C)**, therefore involving peripheral cells and the adaptive immune system. The resulted immune response will in turn affect neuronal integrity and might also enhance further aggregation of a-synuclein in neighboring cells contributing to disease progression. (Image partially constructed using Servier Medical Art).

FcγR also seems to mediate a-syn clearance and internalization into phagosomes, with subsequent nuclear translocation of NF-κB p65, through an IgG independent-internalization (Cao et al., 2012; **Figure 1**). When complexed with antibodies, a-syn aggregates are also internalized through FcγR receptors and clustered in lipid rafts through a more efficient secondary pathway that the one used in the absence of the antibody (Bae et al., 2012). Another group also showed that antibody mediated uptake is more efficient and goes through FcγRI and FcγRIIB/C (Gustafsson et al., 2017). In PD patients, FcγRI and III are expressed on activated microglia or on lymphocyte-like cells, respectively, that are found in the proximity of neurons that presented IgG deposition (Orr et al., 2005), however, the FcγRIIB was absent in the PD brains in that cohort. The FcγRIIB (CD32B) is a low affinity receptor that binds immune-complexed IgG. It is the only inhibitory Fcγ and possess an immunoreceptor tyrosine-based inhibitory motif (ITIM) in its cytoplasmic domain. FcγRIIB cross-linking by immune complexes results in ITIM phosphorylation and inhibition of the activating signaling cascade (Smith and Clatworthy, 2010). FcγRIIB expression in microglial cells during chronic infection, seems to occur to prevent hyper-activation and subsequent brain damage (Chauhan et al., 2017). Aggregated a-syn binds to FcγRIIB on microglia, inducing SHP-1 activation, and inhibiting microglial phagocytosis (Choi et al., 2015). Thus the type and complexes formed by a-syn will significantly change the consequence of its interaction with microglia receptors. Interestingly, FcγRIIB, through downstream mechanism with SHP-1/2, has been suggested to be involved in the propagation of a-syn in neurons (Choi et al., 2018), but this study focused on the expression of the proteins in neurons, rather than microglia. Also, another immune related protein: lymphocyte activation gene 3 (LAG3), has been suggested to be involved in the propagation process, again through expression in neurons (Mao et al., 2016). Equally, as mentioned above, the immune receptor TLR-2 has been also related to a-syn effect in neurons (Dzamko et al., 2017). Consequently, proteins typically related to immune system are being proposed to act as a-syn receptors and/or mediators of the a-syn effect in neurons, therefore, upon a-syn interaction these membrane proteins will initiate a cascade of events both in neurons and in immune cells, and both processes will contribute to the disease progression.

## Activation of Microglia by Alpha-Synuclein and the Receptors Involved

There are now multiple studies showing that extracellular a-syn induces a pro-inflammatory response in microglia with elevated cytokine production such as IL-1β, IL-6, and TNF-α (Klegeris et al., 2008; Su et al., 2008; Lee et al., 2009, 2010; Roodveldt et al., 2010; Couch et al., 2011), increased COX-2 and iNOS (Su et al., 2008, 2009; Lee et al., 2009); and production of free radicals (Su et al., 2008; Lee et al., 2010); a microglia response that resulted in cell toxicity (Zhang et al., 2007; Klegeris et al., 2008). Not only microglia-like cells but also macrophages are activated by monomeric a-syn, inducing TNF-α, iNOS and COX-1 increase, an ability that required both N and C terminal a-syn, but not the NCA region (Lee et al., 2009). The relevance of the aggregation of a-syn has been addressed and several labs confirmed the inflammogen capacity of oligomeric or aggregated/fibrillar a-syn that leads, similarly, to increased TNF-α (Reynolds et al., 2008a; Beraud et al., 2011; Codolo et al., 2013) and ROS (Zhang et al., 2005; Thomas et al., 2007; Freeman et al., 2013), which were also related to cell toxicity (Reynolds et al., 2008a). And comparative studies on the ability of fibrillar, oligomeric and monomeric, side by side, suggest a higher capacity of the fibrillar a-syn compared to the other two types to increase TNF-α and IL-1β release (Hoffmann et al., 2016). In addition, the relevance of the disease related mutation (A53T, A30P, and E46K) has also been addressed with contradictory results (although incubation time and concentration of a-syn in both studies were drastically different). One study reported A30P and E46K a-syn as those with the highest pro-inflammatory ability and almost no effect for the A53T (Roodveldt et al., 2010), but a second group suggested that first A53T and secondly the A30P were those with the highest ability to activate microglia (Hoenen et al., 2016).

One of the first report on the a-syn ability to induce a pro-inflammatory phenotype in microglia was reported back in 2005; Zhang and coworkers showed that oligomeric a-syn was phagocyted by microglia and it led to PHOX NADPH activation (ROS production) and increased Prostaglandin E2 (Zhang et al., 2005). A-syn induced microglia activation led to neuronal toxicity via ROS production through p67 and p47^phox^ membrane translocation and activation of PHOX NADPH oxidase, (NOX2) (Zhang et al., 2005; Jin et al., 2007; **Figure 1**). They reported that deletion of the Prostaglandin E receptor subtype 2 lead to increase microglia ability to degrade a-syn (Jin et al., 2007; Liu et al., 2007), although it did not completely avoid microglia activation, which was partially mediated by CD11b and did not required a-syn internalization (Zhang et al., 2007). Indeed, a-syn interacted directly with CD11b, and this interaction was responsible of the NOX2 activation, which also mediated the chemoattractant ability of a-syn (Wang S. et al., 2015). Interestingly a-syn (29–40) peptide can bind the catalytic subunit gp91 of NOX2, thus inducing H_2_O_2_ production, which in turn activates Erk1/2 kinase that phosphorylates p67 and p47^phox^, which further amplifies the NOX2 response (Wang S. et al., 2016). A recent work further corroborates the relevance of CD11b (α-chain of integrin αMβ2) and suggest that it mediates aggregated a-syn-induced NOX2 activation through a RhoA-dependent pathway (**Figure 1**). In this study, the authors confirmed that a-syn induced membrane translocation of NOX2 cytosolic subunit p47^phox^ and the induction of superoxide production (Hou et al., 2018).

Another membrane receptor associated to a-syn is the scavenger receptor CD36 (through ERK phosphorylation), as its ablation resulted in a decrease of microglia activation and TNF-α release induced by monomeric a-syn (Su et al., 2008) or a double mutant A30P, A53T a-syn (Su et al., 2009; **Figure 1**). The protease activated receptor 1 (PAR-1), a G-protein couple receptor, has been indirectly implicated in the a-syn-induced activation, through a paracrine-autocrine event initiated by the secretion of matrix metalloproteinases (Lee et al., 2010). Also, the purinergic receptors have been implicated in a-syn microglia activation. In BV2 cells, oligomeric a-syn stimulated the microglial P2X7 receptor, with PI3K/AKT activation and increased oxidative stress, via p47^phox^ translocation (PHOX activation) (Jiang et al., 2015; **Figure 1**).

CD14 has also been suggested to mediate monomeric a-syn signaling in microglia since glimepiride, a sulfonylurea that induces release of the CD14-GPi-anchored protein, reduced a-syn microglia activation by decreasing the translocation of TLR-4 into rafts through CD14 (Ingham et al., 2014). CD14, considered the main receptor for LPS, is a co-receptor of the TLR-4 but it has also been related to TLR-2 signaling (Kitchens, 2000; Muroi et al., 2002). These two receptors have been the focus of research in two labs in the field: While Stefanova et al., has been studying TLR-4, Lee et al., had a special focus in TLR-2. Stefanova showed that the lack of TLR-4 *in vivo* was related to increase neurotoxicity and elevated levels of a-syn, decrease of phagocytic ability, increase TNF-α and a pro-inflammatory reaction (Stefanova et al., 2011) suggesting that TLR-4 mediates a-syn phagocytosis. Moreover, *in vivo* treatment with TLR-4 agonist leads to protection in a transgenic a-syn overexpressing line (Venezia et al., 2017). *In vitro*, using BV2 cells and recombinant a-syn, the lab showed that both monomeric and also modified (truncated or fibrillar) a-syn phagocytosis is mediated by TLR-4, through a NF-κB nuclear translocation, which induced releases of TNF-α, IL-6, and CXCL1 (Fellner et al., 2013; **Figure 1**). However, the team noted that the lack of TLR-4 did not completely abolished the inflammatory reaction *in vivo*, implying parallel TLR-4 independent processes (Stefanova et al., 2011).

In parallel, Lee and co-workers has related immune changes in microglia mostly, but not uniquely, to TLR-2. Their work has highlighted the relevance of the a-syn conformation and our ability/limitation to mimic it using *in vitro* recombinant proteins. By means of conditioned media from SH-SY5Y overexpressing a-syn they showed activation of microglia *in vitro* mediated by p-p38 and that was lost if they use microglia lacking TLR-2 but not with TLR-3 or 4 deficient microglia (Kim et al., 2013). Moreover, while *in vivo* overexpression of a-syn in WT mice induced MHCII upregulation in microglia, this was not observed in TLR-2 KO mice, where the correlation between the expression of a-syn and MHC II found in the WT, was lost. Remarkably, this TLR-2 activation was conformational dependent as it was associated to oligomeric a-syn and not monomeric or fibrillar a-syn (Kim et al., 2013; **Figure 1**). However, both labs noted that not all a-syn induced effect on microglia were dependent in their TLR of choice and as listed above, other proteins such as CD11b are also involved in the a-syn activation of microglia, thus resulting in a complex event.

## Cascades Initiated by Alpha-Synuclein in Microglia

### NF-κB

Regarding to the intracellular cascade initiated by a-syn, NF-κB has been consistently implicated in studies with monomeric, oligomeric, aggregated or nitrated a-syn in rodent cell lines and human microglia (Klegeris et al., 2008; Reynolds et al., 2008b; Wilms et al., 2009; Lee et al., 2010; Couch et al., 2011; Hoenen et al., 2016). This could be a consequence of the a-syn interaction with TLR, which leads through the adaptor protein Myeloid Differentiation primary response gene-88 (Myd-88) to activation of the “canonical” IκB kinases, initiating a phosphorylation cascade that results in translocation of NF-κB (**Figure 1**). Indeed, oligomeric, but not monomeric, a-syn induces a pro-inflammatory phenotype in microglia through interaction with a heterodimer TLR-1/2, leading to NF-κB nuclear translocation and increase of TNF and IL-1β, in a MyD88-dependent manner (Daniele et al., 2015; **Figure 1**). Interestingly, the MyD88 cascade leads to phosphorylation of LRRK2; another PD related protein that seems to be also an important player in the immune response (Dzamko et al., 2012). A-syn overexpression increased microglia expression of LRRK2 CD68 and iNOS (Daher et al., 2014). Accordingly, overexpression of a-syn via viral vectors in LRRK2-KO rats resulted in reduced nigral degeneration and decreased number of ameboid microglia expressing CD68 and/or iNOS (Daher et al., 2014). Furthermore, lack of LRRK2 in microglia resulted in an improved ability to clear extracellular a-syn, which was associated to an increase of early endosomes (Maekawa et al., 2016). Thus, suggesting that LRRK2 has a role in the clearance of a-syn and the a-syn induced activation of microglia. However, the role of LRRK2 seems to be more significant due to its putative function not only in microglia, but also in other immune cells, including macrophages, dendritic cells and B cells (Dzamko, 2017).

The signaling cascade involved in the microglia response to a-syn is complex and seems to involve not only NF-κB but also other parallel cascades. Incubation with oligomeric or monomeric a-syn leads to activation of erk1/2 and p38MAPK (Su et al., 2008, 2009; Wilms et al., 2009). The oligomeric a-syn induced TLR2 signaling was mediated by both: NF-κB and p38 (Kim et al., 2013), and p38 and JNK have previously been related to a-syn toxicity and inflammation (Klegeris et al., 2008; Wilms et al., 2009; Prabhakaran et al., 2011). Accordingly, genetic deletion of ASK1 (a MAPK3 that acts upstream of p38) in an a-syn transgenic mice, decreased microgliosis and improved the motor phenotype, although no changes in the a-syn pathology were observed (Lee et al., 2014).

### Inflammasome

As suggested by the increased IL-1β induced by a-syn in microglia, several studies have addressed the involvement of the inflammasome in the a-syn related immune response. In human monocytes aggregated a-syn (40 nM) induced release of IL-1β in a phagocytosis dependent event, which required caspase-1 activation and involved the NLRP3 inflammasome (Codolo et al., 2013). However, another group, did not see this using only a-syn, but if the aggregated a-syn (0.6–2.4 μg/ml, 40–160 nM) was used in LPS-primed THP-1 (a human monocytic cell line), this lead to IL-1β release through a caspase-1 activation, suggestive of inflammasome (**Figure 1**). This disparity might reflect differences in aggregate preparation (2 weeks shaking vs. 3 days, respectively), the time of incubation (6 h vs. 24 h) or the cell used (Freeman et al., 2013). A third group using BV2 cells showed that both monomeric and aggregated WT and A53T a-syn (0.1, 1, and 10 μg/ml) activated caspase-1, p65 nuclear translocation and inflammasome. This effect was endocytosis dependent and mediated through lysosomal damage, cathepsin B release and AMPK-phosphorylation-dependent ROS accumulation (Zhou et al., 2016; **Figure 1**). Interestingly, inflammasome related caspase-1 activation leads to the truncation of a-syn and generation of a pro-aggregatory form of truncated a-syn (1–121) that promotes aggregation and neuronal toxicity (Wang W. et al., 2016).

### Nfr2

Besides the a-syn induced cascade resulting in ROS, the redox status modulates in turn the microglia activity by modifying not only pro-inflammatory transcription factors, such as NF-κB, but also the antioxidant transcription factor Nrf2 (Rojo et al., 2014). Accordingly, monomeric A53T a-syn (but not wild type) induced microglia activation through a phosphorylation mechanism mediated by MAPKs and successive NF-κB/AP-1/Nrf2 pathways activation (Hoenen et al., 2016). Aggregated a-syn induces a robust classic pro-inflammatory status with oxidative stress shown by the increased NO production and elevated NOX1. However, when a-syn is complexed to dopamine this fails to mount similar robust pro-inflammatory response but is able of inducing Nrf2 which in turn induces up-regulation of antioxidant heme oxygenase-1 (HO-1) and the reduction of NOX expression (Beraud et al., 2013). Therefore, the type of a-syn presented by the neuron will be determinant in the microglia response. The relevance of Nfr2 is also highlighted by *in vivo* studies, since lack of Nrf2 resulted on increased a-syn toxicity due to the failure of the microglia to respond, which lead to increased IL-6, IL-1β, iNOS, and reduced phagocytosis, which correlated to a-syn accumulation (Lastres-Becker et al., 2012). Accordingly, overexpression of Nrf2 in brain has been shown to be neuroprotective in a-syn based models (Gan et al., 2012). A recent paper suggest that Nrf2 can also act as a cell-autonomous agent by inducing a-syn clearance in neurons (Skibinski et al., 2017).

### MHCII

As mentioned above, an immune marker expression that correlates directly with a-syn neuronal pathology is MHCII; this is true in humans (Croisier et al., 2005), but also in rodents (Sanchez-Guajardo et al., 2010). MHCII is involved in the presentation of antigens to T-cell, specifically CD4-T cells, thus suggesting that adaptive immune system is also involved in the immune response during PD. Supporting this, PD genetic risk variants have been described in the human leukocyte antigen (HLA) gene loci (Hamza et al., 2010; Nalls et al., 2011; Puschmann et al., 2011; Wissemann et al., 2013). In brain, microglia can act as an antigen presenting cell (Almolda et al., 2015). Thus, we could speculate that upon uptake of a-syn by the microglia, the protein can be processed in endosomes and presented to T-cells via MHCII (**Figure 1**). Accordingly, *in vitro*, aggregated a-syn increased T-cell proliferation in a MHC II dependent manner (Harms et al., 2013). We and others have shown that a-syn PD based models showed T-cell infiltration in brain that correlated to microglia proliferation and activation (Sanchez-Guajardo et al., 2010; Harms et al., 2017). Supporting this possibility, a-syn derived peptides are recognized by T-cells derived from PD patients (Sulzer et al., 2017). Interestingly, a-syn toxicity was abolished in mice in the absence of MHCII, however, the MHCII knock-out line used in this study presents a dramatic decrease of the CD4+ T-cell population thus making difficult to draw conclusions (Madsen et al., 1999; Harms et al., 2013). A deleterious role for CD4 in PD has been previously proposed (Brochard et al., 2009). However, the work of Sulzer supports a Th2 response (thus anti-inflammatory) rather than a deleterious response in the T-cells of PD patients (Sulzer et al., 2017). We have shown that allelic variance of *Mhc2ta* related to lower expression MHCII levels resulted in increased a-syn toxicity in rats that correlated with a higher microglia response (Jimenez-Ferrer et al., 2017), thus suggesting that a failure in the adaptive immune signal (MHCII-CD4 T cell) will have consequences in the neuronal survival to a-syn insult. In PD patients the CD4 population seems to be altered (Fiszer et al., 1994; Saunders et al., 2012; Stevens et al., 2012) and they seem to be more prone to apoptosis, and differ in their activation state, which could suggest that a failure of the CD4 cells response might be happening in PD (Romero-Ramos et al., 2014). However, we should also consider a differential response of the T-cells to the different types of a-syn presented through the disease, thus changing the nature of their response as disease progress. Accordingly, we have recently shown that the CD4 response was variant specific (Olesen et al., 2018).

## Limitations of the Studies Performed So Far

Altogether, the literature is now abundant about the character of a-syn as a DAMP, however, certain problems have arisen through the last decade which complicates the comparison of the studies performed in multiple labs. The choice of cellular or animal model, as well as the concentrations and or preparation of the different forms of a-syn is not trivial. For example although BV2 cells are widely used as a microglia model (Blasi et al., 1990), they do not always predict fully the *in vivo* response of microglia and also differ in their response from primary microglia (Henn et al., 2009). In addition, studies using monocytes or monocytic-cell lines, such as RAW 264.7 (murine) and THP-1 cells (human), although relevant for the disease, they will not always translate directly to microglia response, since as mentioned they constitute different cells.

A consensus about preparation of a-syn oligomers or fibrillar aggregates is also important, since changes in the type of a-syn strain used, is determinant in the type of pathology observed in brain (Peelaerts et al., 2015). Also, the presence or absence of endotoxin in preparations of recombinant a-syn and during fibrilization has also been investigated and shown different effects when approached *in vivo* (Rutherford et al., 2015; Kim et al., 2016). Some of these issues have been visited and protocols have been put forward recently with an initiative of the MJ. Fox Foundation (Polinski et al., 2018), an initiative that the scientific community should positively embrace.

Finally, the concentrations used in each experiment is also highly relevant. Although, initial papers studying *in vitro* a-syn effect on microglia were using rather high concentrations, on the range of μM, the recent papers regarding TLR-2 and TLR-4 activation by a-syn are based on much lower although also variable concentrations, 5.3 μg/ml (360 nM) and 3 nM. This is relevant, especially considering that a-syn concentrations in CSF are reported to be 550 pg/ml (35 pM, using a Luminex bead-based assay) (Wang et al., 2012). Furthermore, a-syn levels in brain has been shown to be 2–5 ng/μg protein in healthy humans and up to 17.5 ng/μg in MSA (using calibrated western blots) (Tong et al., 2010). Microdialysis experiments (followed by sandwich ELISA) showed 0.15 ng/ml in wild type mouse and 0.49 ng/ml in a transgenic a-syn line. Moreover, the authors reported also that in humans 0.5–8 ng/ml a-syn were found in brain parenchyma (Emmanouilidou et al., 2011). This is corroborated in a recent paper reporting a concentration of a-syn in the interstitial space of 1–5 ng/ml (68.49–342, 45 pM) using microdialysis in mice (Yamada and Iwatsubo, 2018). The levels of extracellular a-syn in cell culture are reported to be higher, in the range of 2–12 nM (Emmanouilidou et al., 2010). But conditioned media containing as little as 1.06 ± 0.371 μg/ml (70 nM) oligomeric a-syn, has been shown to have an effect in microglia TLR-2 (Kim et al., 2013). Thus, the relevance of some of the studies reviewed here, should be put in context, with respect to the physiological significance. Although, we could speculate that high concentration of the a-syn could be achieved locally at the level of synapse or high neuronal activity areas, since as suggested, a-syn release increases with neuronal activity (Yamada and Iwatsubo, 2018).

## Neuroprotective Strategies Based on Immunomodulation

With the knowledge gained through the last decade, several immune-based therapeutic approaches have been recently tested. Several laboratories have investigated a-syn toxicity in the viral vector PD based model in several knock-out lines and suggested protective roles for fractalkine (Thome et al., 2015), highlighted the key inflammatory role of the microRNA155 (Thome et al., 2016) and the role of TLR2 in a-syn toxicity (Kim et al., 2013). Although, knock-out studies constitute a useful proof of principle approach, the *in vivo* pharmacological modulation of the a-syn induced immune cascade will have a more translational significance.

In that regard, several recent studies have approached drugs and natural compounds that can interfere with relevant immune related cascades to achieve neuroprotection. Pharmacologic inhibition of the JAK1/2, was protective in the a-syn viral vector model *in vivo* (Qin et al., 2016). MAPK inhibitor semapimod, also protected against a-syn induced microgliosis and dopaminergic cell death *in vivo* (Wilms et al., 2009). And several studies have approach NF-κB and NOX2 cascade to decrease neurodegeneration. Taurine, a major intracellular free β-amino acid in mammalian tissues, was neuroprotective in mice injected with paraquat and maneb (Che et al., 2018). Taurine interfered with the NF-κB pathway and the membrane translocation of p47^phox^ (thus NOX2 activation), and it supressed paraquat and maneb-induced microglial M1 polarization. This resulted in decreased dopaminergic neurodegeneration and a-syn aggregation (Che et al., 2018). Equally α-mangostin, a polyphenolicxanthone from mangosteen, also inhibited NOX2 and NF-κB signaling induced by a-syn in culture, thus supressing microglial release of pro-inflammatory cytokines, iNOS and ROS production (Hu et al., 2016). Lenalidomide is a thalidomide derivative with reduced toxicity that when administrated to transgenic a-syn Thy1- mice improved motor performance abilities and attenuated dopaminergic degeneration and microgliosis, by modulating NF-κB signaling and cytokine expression (Valera et al., 2015). NOX2 inhibitor diphenyleneiodonium treatment in several PD mouse models was neuroprotective (Wang Q. et al., 2015). When LPS was used in A53T a-syn transgenic, diphenyleneiodonium could protect neurons by avoiding pro-inflammatory activation of microglia, reducing oxidative stress and it lead to a decrease of a-syn accumulation (Wang Q. et al., 2015).

Dimethyl fumarate is a potent anti-inflammatory and anti-oxidant fumaric acid esters. On the MPTP mouse model, dimethyl fumarate significantly reduced a-syn aggregates (dimers and oligomers) rescuing neurons from oxidative stress via activation of the Nrf2 transcriptional system, suppression of NF-κB signaling and consequently decreasing COX-2 and IL-1β levels (Campolo et al., 2017). In the same model and on a similar way of action, the novel bibenzyl compound 2-[4-hydroxy-3-(4-hydroxyphenyl) benzyl]-4-(4-hydroxyphenyl) phenol, was neuroprotective not only by regulating NF-κB signaling, but also by supressing NOD-like receptor protein 3 (NLRP3) inflammasome pathway (Zhang et al., 2017).

Another natural compound, Juglanin (from Polygonum aviculare) was neuroprotective in mice treated with LPS, by supressing LPS-induced inflammation through the TLR-4/CD14/MyD88 pathway and thus decreasing the release of pro-inflammatory cytokines (IL-1β, TNF-α, IL-18) and COX-2 (Zhang and Xu, 2018). Interestingly, as an indirect effect, LPS induced increased on a-syn mRNA was reversed by Juglanin in a dose dependent manner. Thus, although several of these studies were not using a-syn based models, the mechanism of neuroprotection could also be relevant in a-syn induced neurodegeneration.

In parallel, enhancement of a-syn clearance is the therapeutic strategy approached by other labs. Stefanova’s team aimed to increase a-syn uptake through TLR-4 using monophosphoryl lipid A, a TLR-4 selective agonist and a potent inducer of phagocytosis. The drug was neuroprotective in MSA model, and chronic treatment reduced the a-syn accumulation, neuroprotection and improved motor behavior (Venezia et al., 2017). Other labs have approached passive immunization to facilitate a-syn degradation and found neuroprotection in multiple a-syn based models (Bae et al., 2012; Games et al., 2014; Mandler et al., 2015; Spencer et al., 2017). With that purpose, novel antibodies have also been generated against specific peptides (targeting the N-terminal or central region of α-synuclein-AB1 and AB2 respectively) on full-length human a-syn and tested on a viral vector-based a-syn model in rats. Both showed beneficial neuroprotective effects, but particularly AB1 demonstrated to be more efficient (Shahaduzzaman et al., 2015). Additionally, passive vaccination with a a-syn antibody that has preference for aggregated a-syn, is being approached in a Phase 1 trial by Prothena in collaboration with Hoffman-La Roche^1^.

Interestingly, a-syn vaccination also results in neuroprotection in the MPTP toxic PD model (Villadiego et al., 2018). An innovative vaccination strategy that combines a-syn and Glucose related protein 94(Grp94), a chaperone protein found in the endoplasmic reticulum with critical functions in physiology and development of multicellular organisms (Marzec et al., 2012), demonstrated a strong disease-modifying potential with the ability to target neuroinflammation in the MPTP model (Villadiego et al., 2018). In the same model, while vaccination with nitrated a-syn induced a Th17 response and neurodegeneration, the adoptive transfer of T-regulatory cells, lead to neuroprotection (Reynolds et al., 2010). Thus, suggesting the importance of the type of a-syn that the immune system is recognizing, which is corroborated by our own data showing that the T-cell compartment recognized and responded to the different a-syn and its disease related modifications (fibrillar and nitrated) *in vivo* (Olesen et al., 2018).

We have previously reported that an active a-syn vaccination in the a-syn viral vector PD model, resulted in decreased a-syn pathology that was correlated to the humoral response (antibody production and IgG deposition in brain) but also to a cellular response with increase infiltration of T-regulatory cells in brain (Sanchez-Guajardo et al., 2013). In a follow up study, we showed how the vaccination strategy, was able of modifying the peripheral T-cell compartment, thus corroborating the role of the periphery in the disease and its influence in brain events (Christiansen et al., 2016). The advantages of a combined cellular and humoral response have been recently confirmed in an a-syn transgenic mouse (Rockenstein et al., 2018). An active vaccination in PD patients is being conducted by the company Affiris, and it has initiated its Phase 2 after a successful Phase 1^2^.

## Conclusion

The evidence so far suggest that the microglia will respond to the changes in neurons during PD, it will not only sense changes in neurotransmitter release and or modifications in other proteins expressed by neurons such as CD200 or CX3CL1 (Ransohoff and Cardona, 2010), but it will also recognize and respond to a-syn. This a-syn might be encountered upon trogocytosis of axonal structures, but also in the extracellular milieu upon exocytosis. The type of a-syn will change as the disease progresses, i.e., monomeric, aggregated, nitrated, truncated, and also it will be affected by interaction with other molecules depending on the neuronal population or disease stage. The microglia will recognize, uptake and phagocyte the a-syn, in a process that will be dependent on the type of a-syn encountered and the proteins and cascades involved in such event might differ accordingly (**Figure 1**). For example, aggregated a-syn induced a TNF response in microglia, but upon dopamine modification a-syn does not efficiently initiates such inflammatory cascade but seems to favor Nrf2 expression (Beraud et al., 2013). Or the clearance of a-syn through FcγR seems to be more efficient if complexed with IgG (Bae et al., 2012). In addition, as mentioned before, while TLR-4 recognized and uptakes monomeric a-syn, TLR-2 seems to be especially relevant to recognize an epitope associated to oligomeric a-syn (Kim et al., 2013). However, the research community seems to agree on the pro-inflammatory character of the event initiated by aggregated a-syn, which will in turn affect neuronal integrity and might also enhance further aggregation of a-syn in neighboring cells. In parallel to the local neuronal response, the microglia would also interact with peripheral lymphocytes, that will eventually also modulate the immune response in the patient. Altogether leading to a dynamic response of the immune system in the disease as time progresses. Thus, if aiming for immunomodulation, in the future we should take into account at the status of the immune system in the patient, so appropriate neuroprotective factors are enhanced accordingly. Therefore, a more personalized therapeutic design should be considered that will require a profiling of the patient’s immune system prior to initiation and selection of the pertinent immunomodulatory agent.

## Author Contributions

SF made substantial contributions to the conception of the work, wrote part of the first draft of the manuscript, managed references, prepare the figure, and brought constructive changes to the final text of the manuscript. MR-R conceived, designed, and wrote the manuscript.

## Conflict of Interest Statement

The authors declare that the research was conducted in the absence of any commercial or financial relationships that could be construed as a potential conflict of interest. The reviewers AS and SB and the handling Editor declared their shared affiliation.

## Footnotes

1 http://globenewswire.com/news-release/2015/03/19/716962/10125642/en/Prothena-Reports-Robust-Reduction-of-Free-Serum-Alpha-Synuclein-of-up-to-96-After-Single-Dose-of-PRX002-a-Novel-Protein-Immunotherapy-for-Parkinson-s-Disease.html

2 http://www.affiris.com/news/affiris-announces-results-of-a-phase-i-clinical-study-using-affitopes-pd01a-and-pd03a/
